# The Short Overview on the Relevance of Fatty Acids for Human Cardiovascular Disorders

**DOI:** 10.3390/biom10081127

**Published:** 2020-07-30

**Authors:** Viktoriya S. Shramko, Yana V. Polonskaya, Elena V. Kashtanova, Ekaterina M. Stakhneva, Yuliya I. Ragino

**Affiliations:** Branch of the Institute of Cytology and Genetics, Siberian Branch of Russian Academy of Sciences, Research Institute of Internal and Preventive Medicine, 630089 Novosibirsk, Russia; nosova@211.ru (V.S.S.); yana-polonskaya@yandex.ru (Y.V.P.); elekastanova@yandex.ru (E.V.K.); ragino@mail.ru (Y.I.R.)

**Keywords:** saturated fatty acid, monounsaturated fatty acid, polyunsaturated fatty acid, cardiovascular disease, blood lipid profile

## Abstract

This review presents existing evidence of the influence of saturated and unsaturated fatty acids on cardiovascular diseases (CVD). Data are discussed regarding the roles of the most relevant fatty acids, such as myristic (C14:0), palmitic (C16:0), stearic (C18:0), palmitoleic (C16:1), oleic (C18:1), linoleic (C18:2), *α*-linolenic (C18:3, *ω*-3), *γ*-linolenic (C18:3, *ω*-6), arachidonic (C20:4), eicosapentaenoic (C20:5), docosahexaenoic (C22:6), and docosapentaenoic (C22:5) acid. The accumulated knowledge has expanded the understanding of the involvement of fatty acids in metabolic processes, thereby enabling the transition from basic exploratory studies to practical issues of application of these biomolecules to CVD treatment. In the future, these findings are expected to facilitate the interpretation and prognosis of changes in metabolic lipid aberrations in CVD.

## 1. Introduction

Diseases of the blood circulation system, primarily ischemic heart disease (IHD), rank first in terms of the prevalence of complications and death in Westernized societies, being responsible for one of every three deaths in the United States and one of every four deaths in Europe [1,2]. The 2013 Global Burden of Disease study estimating that cardiovascular diseases (CVD) caused 17.3 million deaths globally. It accounted for 31.5% of all deaths and 45% of all non-communicable disease deaths, more than twice that caused by cancer, as well as more than all communicable, maternal, neonatal, and nutritional disorders combined [2]. In addition to systemic inflammation, oxidative stress, and disruption of lipid metabolism, which are risk factors for the development and progression of atherosclerosis and the related CVD, fatty acid (FA) metabolic abnormalities became also an important risk factor. Much attention is given to the research on FA, with particular emphasis on the their amount and type consumed, and there are studies on potential utility of FA as biomarkers of the functional state of the human body for early diagnosis of CVD and especially atherosclerosis [3,4,5,6,7,8]. In cells of human tissues, ~70 FA have been identified as components in a structure of lipids, with more than a half of these FA detected in trace amounts, a less than 0.1% proportion.

The American Heart Association/American College of Cardiology guideline has recommended to decrease intake of saturated FA (SFA) to 5% to 6% of total daily energy (calorie) intake to reduce the risk of CVD [9]. The scientific rationale for decreasing SFA in the diet has been and remains based on well-established effects to raise low-density lipoprotein (LDL) cholesterol, along with a reduction in non–high-density lipoprotein (HDL) cholesterol, a leading causes of atherosclerosis [10]. On the contrary, polyunsaturated FA (PUFA) are considered to ameliorate lipid markers, with omega-3 PUFA consumption resulting in reduction of plasma triacylglycerols (TG) and ApoB-100, which in turn reduces the concentration of LDL cholesterol [11]. Reducing SFA and replacing it with PUFA in randomized controlled trials has reduced the incidence of CVD which included myocardial infarction (MI) (fatal and non-fatal combined) and IHD events [12,13]. In Finland, a successful nationwide health project to lower the very high rate of IHD mortality, started in 1972, had as a major goal the reduction in the high intake of SFA [14]. Regarding monounsaturated FA (MUFA), the data are more limited, but in vivo studies like those of Macri et al. [15], and Alsina et al. [16], found that olive oil and fish oil, rich in MUFAs, is highly effective in decreasing the oxidization of LDL, and TG levels [17]. In the populations with very low SFA intake have very low rates of CVD [18], and members of many single populations who have low SFA high unsaturated FA intake have lower future incidence of CVD compared with those with high SFA and low unsaturated FA intake [19]. Therefore, the Dietary Guidelines recent years is to shift food choices from those high in SFA to those high in MUFA and PUFA [9,20].

As lipids constitute a major portion of the majority membranes suggest that the presence of massive concentrations of unsaturated FA within membranous structures. In addition, well recognized that the PUFA are bioactive mediators of diverse pathways involved in cellular homeostasis or, in some cases, interact with cellular macromolecules resulting in cell death [21]. These cellular responses may be a consequence of the vulnerability of unsaturated FA to diverse oxidation reactions, or radical reactions, or both. Reactive oxygen species readily bind to unsaturated FA in lipids that contain multiple double bonds (DB), “steal” electrons, and trigger a free radical chain reaction. This oxidative process usually consists of initiation (production of a FA radical), propagation (creation of a peroxyl-FA radical), and termination (production of electrophilic carbonyls [22,23]. The free radical mediated production of electrophilic products of PUFA proceeds by autocatalysis and is, as a result, not well regulated. Thus, free radical mediated lipid peroxidation is more commonly associated with diseases of sustained oxidative stress including atherosclerosis and the related CVD [24].

In the past few years researchers have come to discordant conclusions about the relationship between dietary FA and risk of CVD [12,13,20]. This has created confusion among patients, their physicians, and the public. The objective of this review is to present of results regarding effect most relevant FA on CVD, so that understand the reasons for the divergent findings.

## 2. Saturated Fatty Acids (SFA)

Among FA, the structure of SFA is the stability and their physical properties depend on their molecular weight (Table 1).

SFA are resistant to oxidation. The cellular membranes and lipoproteins containing large amounts of SFA are less active functionally. Such lipoprotein particles form stable bonds with cellular receptors of lipoproteins, thus promoting disorders of the cholesterol transport system in the human body and leading to the development of the dyslipoproteinemias that contribute to atherosclerogenesis: So-called atherogenic dyslipoproteinemias [25,26].

It is believed that consumption of food with SFA is associated with increased risk of CVD according, which is mediated by increased levels in serum of total cholesterol by increasing cholesterol levels LDL cholesterol. A number of studies have shown that increased consumption of SFA is associated with both an increased incidence of IHD and the severity of atherosclerotic lesions of the arteries [13,27,28,29,30,31,32].

A recent meta-analysis, which included 15 randomized controlled trials involving over 59,000 people, assessed the effect of reduced dietary the amount of SFA on mortality and cardiovascular morbidity [13]. This systematic review suggests that reducing SFA in the diet for at least two years reduced MI and IHD events, but no effects on IHD mortality, non-fatal MIs, or stroke. This clear effect on cardiovascular events was not lost on sensitivity analyses. The reduction in cardiovascular events was clearer in subgroups with greater baseline SFA intakes, greater reduction in SFA in the intervention group, and studies with greater serum total cholesterol and LDL cholesterol reductions. Meta-regression confirmed that degree of reduction in cardiovascular events was related to degree of reduction of serum total cholesterol, and there was a modest suggestion of greater protection with greater SFA reduction or greater increase in unsaturated FAs in the diet.

**Palmitic acid (C16:0)** is one of the important constituent acids of TG in adipose tissue. In this regard, palmitic acid content of blood has the greatest diagnostic and prognostic significance. It is considered that palmitic acid is linked with adverse cardiovascular events [27,28], and its high consumption raises the risk of CVD. The results of the randomized crossover investigation indicate that the palmitic acid-enriched diet resulted in increased fasting plasma LDL cholesterol, and HDL cholesterol concentrations [29]. It has been shown that after a decrease in the intake of SFA, blood concentrations of palmitic acid, LDL, and glucose diminish [30]. Results of a prospective study on US females and males [31] suggest that high blood levels of palmitic FA are associated with a high risk of the onset and progression of IHD. The prospective case-control study CIRCS [32], conducted by Japanese scientists, has yielded similar results. The LURIC study (the Ludwigshafen Risk and Cardiovascular Health study) [33] have investigated the link of SFA in the blood with overall and cardiovascular mortality among patients referred to coronary angiography. The results revealed that palmitic acid is related to a higher risk of death from CVD. Palmitic FA, by enhancing inflammation-related signaling of lipopolysaccharides in macrophages, promotes inflammation and the development of CVD [34,35,36]. In addition, inflammatory activity probably is characterized by increased production of pro-inflammatory cytokines and oxidants, leading to cellular hypertrophy and apoptosis. The findings show that elevated levels of palmitic acid and likely other SFAs can contribute significantly to cardiac damage [37].

**Myristic acid (C14:0).** In tissues of humans and animals, this acid is present at relatively low concentrations, on average, 1% of all FA by weight [38]. Although myristic acid is a minor plasma SFA, it has attracted growing attention because of clinical evidence suggesting its potent cholesterol-upregulating action [39]. Therefore, Fattore E. et al. [40] conducted a systematic meta-analysis, comprising a total of 51 studies with the participation of 1526 volunteers. The results of this meta-analysis show that the major dietary saturated fats (palmitic, stearic, lauric, and myristic acids) have differential effects on the lipid profile: Myristic and lauric acids increase all the cholesterol fractions (e.g., total cholesterol, LDL cholesterol, HDL cholesterol, LDL cholesterol/HDL cholesterol ratio, TG, apolipoprotein A-I, and apolipoprotein B) more than does palmitic acid, and palmitic acid increases all the cholesterol fractions more than does stearic acid. In a prospective case-control study known as CIRCS [32], blood levels of myristic acid were higher in patients with IHD than in a control group of subjects. Multivariate regression analysis uncovered a link of myristic FA with higher IHD risk.

**Stearic acid (C18:0).** In contrast to palmitic acid, which correlates with hypercholesterolemia (HC), it is considered that stearic SFA does not have a significant influence on lipid metabolism [41]. However, in the randomized investigations Meng H. et al. [29] and Mah E. et al. [42] the stearic acid-enriched diets resulted in lower fasting plasma LDL, HDL, and non–HDL-cholesterol concentrations. By contrast, in the randomized controlled trial Baer D. J. et al. [43] and in a study by Mensink R.P. et al. [10] consumption of stearic acid as an supplement did not affect the in blood lipids and any of the primary risk factors for cardiovascular disease. In general, the data on the influence of stearic acid on CVD are contradictory. For instance, in the prospective investigations [44,45], it has been demonstrated that high consumption of stearic acid does not correlate with a higher risk of IHD and MI. By contrast, in the prospective cohort study by Zong et al. [46] and in research Praagman et al. [47], stearic acid was found to make a major contribution to the development and course of IHD. Hunter et al. [48] have shown an independent relation of stearic FA with a higher risk of IHD, but this link turned out to be weak after normalization to the sum of other studied SFA (lauric, myristic, and palmitic acids). Harvey et al. [27] have reported that stearic acid induces apoptosis and necrosis of endothelial cells more strongly than does palmitic or myristic FA. Furthermore, those authors advanced a hypothesis that intracellular accumulation of stearic FA can be proinflammatory and lipotoxic.

Epidemiological research indicates that SFA, especially those containing 12–16 carbon atoms, have the greatest effect on the blood concentration of LDL cholesterol and therefore are often associated both with a higher risk of CVD and with the severity of atherosclerotic lesions in arteries [38,49,50,51]. Present dietary guidelines recommend keeping SFA intake at 8–10% of total energy intake for the prevention of IHD and the reduction of SFA consumption and increase of PUFA consumption is the most efficient method for the normalization of the lipid value in the blood. A change in the proportions of the ingested-with-food FA that affect the ratio of HDL cholesterol to LDL cholesterol may be more important than the simple limiting of SFA, at least myristic and stearic ones, both of which influence the HDL cholesterol level [52].

Clinical trials offer conflicting conclusions regarding the role of SFA and the risk of IHD and its clinical complications. But growing number of studies indicate that the impact of SFA on the course and mortality rates of CVD, is not so much dependent on the overall amount of SFA in the human body but rather on their ratio to unsaturated FA. A recent Cochrane meta-analysis [13] showed moderate-quality evidence that replacing the energy from SFA with PUFA reduces the risk of CVD events and MI, but no effect on all-cause mortality or IHD mortality.

## 3. Unsaturated Fatty Acids

### 3.1. Monounsaturated Fatty Acids (MUFA)

The FA that has a pair of hydrogen atoms missing in the middle part, and the freed bonds are connected to double bond between carbon atoms are called monounsaturated FA (Table 2).

Lately, there is growing interest in investigating the participation of MUFA in the development of CVD because prospective studies have yielded inconsistent results about the influence of MUFA on IHD risk. It is known that MUFA are involved in many physiological processes in the human body, including energy metabolism, lipid biosynthesis, the maintenance of membrane integrity, antioxidant reactions, apoptosis, and aging [53,54]. Among MUFA, palmitoleic (C16:1) and oleic (C18:1) acids are most important in terms of the functional role in the human body.

**Palmitoleic acid (C16:1)** is one of the main MUFA of the omega-7 (*ω*-7) FA family. There is evidence that the C16:1 acid is a major product of endogenous lipogenesis [55]. In the human body, the biosynthesis of *cis*-palmitoleic acid proceeds mainly in the liver and to a lesser extent in adipose tissue, where this FA is later incorporated into phospholipids, TG, waxes, and cholesterol esters [56].

Epidemiological studies suggest that palmitoleic FA participates in cholesterol metabolism and hemostasis, but its influence on the cardiovascular system is inconsistent. In some randomized controlled trials, consumption of capsules or diets rich in palmitoleic acid has been associated with lower blood concentrations of LDL cholesterol and TG and with substantial upregulation of HDL cholesterol [56,57]. Other studies have uncovered correlations between palmitoleic FA and such cardiovascular risk factors as hypertension; high blood levels of total cholesterol, TG, apolipoprotein A1, and apolipoprotein B; and endothelial dysfunction. Palmitoleic acid may behave as an SFA rather than a MUFA by increasing insulin resistance and heart rate and can affect the levels of total cholesterol and LDL cholesterol [58,59]. One research group [59] has found it intriguing that palmitoleic (C16:1) acid can raise the LDL cholesterol level more strongly than palmitic acid (C16:0). Chei et al. [32] have demonstrated that C16:1 acid content not only was higher in a group of patients with IHD than in a control group but also increased the risk of IHD. Overall, the discrepancies in the results may derive from differences in study populations. Nonetheless, most investigators tend to believe that for subjects with definite dyslipoproteinemias, palmitoleic acid as a nutritional supplement can be the main intervention for improving the blood lipid profile [60,61].

**Oleic acid (C18:1)** is the most prevalent MUFA in human food. It constitutes approximately half of FA content of TG in adipose tissue and has turned out to be the main acceptor of reactive oxygen species (ROS) in models of oxidative stress. The strongest oxidants for oleic FA are the superoxide anion-radical, nitrogen dioxide, and ozone [62]. When they act on the C18:1 acid, hydroxy derivatives [63] and/or short-chain SFA are formed. Research on oleic acid has revealed its influence on the cardiovascular system [64]. Oleic acid can improve the blood lipid profile [65], maintain healthy body weight [66], and even prevent palmitic-SFA-promoted mitochondrial dysfunction, insulin resistance, and inflammation-related signaling in neuronal cells [67] and skeletal muscle [68]. Nevertheless, the molecular mechanisms responsible for the protective effects of oleic acid against CVD are poorly studied.

Enrichment of LDL particles with oleic acid can shorten the stay of LDL particles in the artery wall and consequently may lower the risk of atherosclerosis [69]. Harvey et al. [70] propose that endothelial cells attempt to turn excessively taken up stearic acid into the more beneficial oleic acid. A positive correlation between blood levels of oleic acid and TG has been noted by Duarte et al. in patients with heart disease [71]. Those authors theorized that high levels of oleic acid can cause initial events of the atherosclerotic process. Delgado et al. [36] have discovered a direct link of oleic acid with markers of inflammation and with heart failure.

Thus, further research is needed on the impact of MUFA on IHD risk factors and clinical endpoints in order to elucidate a possible role of MUFA in primary and secondary prevention of IHD.

### 3.2. Polyunsaturated Fatty Acids (PUFA)

In Table 3 shows two main families: omega-3 (*ω*-3) and omega-6 (*ω*-6) PUFA, depending on the position of the first double bond (if we count from the methyl end of the group).

## 4. Omega-3 Polyunsaturated Fatty Acids

Long-chain *ω*-3 PUFA represent a special class of FA that contain multiple DB, with the last DB being located three carbons away from the methyl terminus of the chain [72]. Abundance of *ω*-3 PUFA is good for health, e.g., for the treatment of neurological problems [73], symptomatic relief of inflammatory disorders [74], improvement of whole-body metabolism [75], and for reducing the risk of CVD [72].

In the last years, many investigations have focused on the effects of *ω*-3 PUFA in the prevention and treatment on major cardiovascular events. A recent Cochrane meta-analysis, which included 79 randomized controlled trials involving over 112,000 people, suggests that long-chain omega-3 FA do not have important positive or negative effects on mortality or CVD events and that they have little or no effect on other measures of cardiovascular health in primary or secondary prevention [76]. There was a suggestion that long-chain omega-3 FA reduced IHD events. However, this was not maintained in sensitivity analyses because all evidence was of moderate GRADE quality [76].

Another meta-analysis was conducted of 10 long-term, randomized trials of *ω*-3 FA as either primary or secondary prevention in a total of 78,000 participants. During the entire observation period of meta-analysis there was no evidence of significantly lower rates of IHD or major vascular events among the patients who had received *ω*-3 FA than among those in the control groups [77].

The recommendations to increase *ω*-3 PUFA intake are based on observational evidence, but the randomized trials of supplements have largely not shown cardiovascular benefit.

In the randomized Studies ASCEND (A Study of Cardiovascular Events in Diabetes) [78] and VITAL (The VITamin D and OmegA-3 TriaL) [79] evaluated the efficacy and safety of daily supplementation with *ω*-3 FA, as compared with placebo, in relation to the risk of serious vascular events. But the trials have not suggested any differences in the effects of *ω*-3 FA supplementation in the endpoints of major cardiovascular events.

However, when considering each *ω*-3 PUFA can observe its own patterns.

***α*-Linolenic acid (ALA) (C18:3)** belongs the family of essential *ω*-3 PUFA and contains three DB at positions 9, 12, and 15. ALA is an essential FA, an important part of a mixed diet, predominantly found in plant oils, such as flaxseed and rapeseed oils. The findings in a recent Cochrane meta-analysis, which included 79 randomized controlled trials, found that increasing ALA probably slightly reduces risk of IHD mortality and arrhythmia, and may slightly reduce risk of CVD events [76]. According to a meta-analysis by Pan et al. [80], consumption of ALA probably can benefit the cardiovascular system, and each 1 g/day increase in ALA intake is associated with a 10% decline in the risk of death from IHD. In a double-blind, randomized controlled clinical trial by Bloedon et al. [81] it was found that higher consumption of flaxseed oil can short lived LDL-cholesterol lowering effect, lipoprotein(s) and HDL. The suppressive effect of ALA on blood levels of total cholesterol, LDL and TG concentrations has been demonstrated in a randomized controlled study where walnuts and fatty fish served as a food supplement [82]. There are significant inverse correlations between the blood concentration of ALA and intima-media thickness of the internal carotid artery and between the blood docosahexaenoic acid (DHA) level and intima-media thickness of the common carotid artery [83,84,85]. In a small crossover trial with participants who had their first MI, the average intima-media thickness of the carotid artery was also inversely related to ALA [86]. Lemaitre et al. [87] have stated that the risk of sudden cardiac arrest increases with increasing ALA levels. There is a need for more prospective studies for clarifying the link between ALA and diseases of the circulation system, but some authors believe that ALA has moderate protective effects on the cardiovascular system [88].

**Eicosapentaenoic acid (EPA) (C20:5)** is one of the main components of complex lipids and is an essential PUFA. The mechanism behind the influence of EPA on atherosclerosis development consists of effects on endothelial dysfunction and oxidative stress as well as increased synthesis of eicosanoids (which dilate blood vessels and reduce thrombogenesis and inflammation), alleviation of atherogenic dyslipoproteinemia, and other effects [89]. Results from the large prospective randomized clinical trial JELIS [90], showed that administration of EPA 1.8 g/day resulted in a 19% reduction in cardiovascular events in statin-treated patients and a decrease in blood concentration of LDL by 25% after the treatment. Unstable angina pectoris and coronary phenomena were also significantly alleviated in the group of patients receiving EPA. The researchers concluded that EPA is a promising FA for the prevention of major coronary events, especially nonfatal ones, in patients with HC.

At present, the best available evidence for a role of omega-3 fatty acids in atherosclerotic cardiovascular disease risk reduction is REDUCE-IT (the Reduction of Cardiovascular Events with Icosapent Ethyl–Intervention Trial) [91]. The Icosapent ethyl is a highly purified and stable EPA ethyl ester. The results of REDUCE-IT provide evidence that the patients with elevated triglyceride levels who received 2 g of icosapent ethyl twice daily relative to risk of major ischemic events, including cardiovascular death, was significantly lower.

EPA is beneficial for endothelial function: In human umbilical vein endothelial cells (HUVECs), EPA improves the balance between nitric oxide and peroxynitrite and acts synergistically with statins [92]. EPA attenuates palmitic-acid-induced formation of ROS, expression of adhesion molecules and cytokines, activation of apoptosis-related proteins, and apoptosis of HUVECs [93,94]. In addition, EPA inhibits lipid peroxidation processes in membrane vesicles [95]. These antioxidant properties may be attributed to the incorporation of EPA into the lipid bilayer, where EPA can prevent the spread of ROS and preserve the structural organization of lipid membranes [96].

It has been hypothesized that EPA induces neovascularization involving human endothelial-cell progenitors, thereby preventing ischemic damage [97]. Because EPA has high lipophilicity, its possible therapeutic effects on the atherosclerotic plaque include anti-inflammatory and antioxidant activities, a reduction in monocyte adhesion to an endothelium, a decrease in the accumulation of macrophages and foam cells in lipid spots, and an increase in the thickness of the fibrotic cover on top of a lipid-rich plaque [89,98,99,100]. Zampelas [101] has determined that the proportion of EPA in phospholipids of stable plaques inversely correlates with inflammation of the plaques and the number of T-cells there. EPA diminishes intima-media thickness of the carotid artery in patients with hypertriglyceridemia and patients with risk factors of atherosclerosis, despite treatment of the main diseases [102,103]. There are data on the ability of EPA to resolve an inflammatory process via the synthesis of resolvins and special proteins [104,105]. Both substances reduce the recruitment of neutrophils from the blood, thus helping to resolve blood vessel inflammatory processes seen in atherosclerosis [106]. Incorporation of EPA into the membranes of platelets may reduce their aggregation, and in case of a rupture of an atherosclerotic plaque that leads to acute coronary syndrome, EPA may help to limit the size of the adjacent clot by diminishing platelet aggregation, thereby minimizing MI volume [89]. Additionally, EPA not only improves the blood lipid profile but also helps to lower the levels of vascular inflammatory biomarkers, thereby inhibiting the development of clinically important cardiovascular events. The use of EPA as an adjunctive therapy helps to lower the risk of CVD [107].

**Docosahexaenoic acid (DHA) (C22:6)** has unique stereochemical structure, features the highest unsaturatedness, ensures effective signal conductance in neurons thereby preventing spasms of the heart and blood vessels [108], and can have antithrombotic, antiatherogenic, antiarrhythmic, and vasoprotective actions.

Qi et al. [109] have shown that DHA lowers blood TG levels by reducing the activity of liver enzymes. Simultaneously, there was an increase in blood HDL levels, through enhanced synthesis of phospholipids. Honda et al. [110] and Wang et al. [111] have demonstrated that DHA lowers such inflammation markers as interleukin 1-β, tumor necrosis factor *α* (TNF-*α*), and interleukin 6. There is evidence that the blood level of DHA is linked with the endothelial function in patients with IHD. These results imply that that a low DHA concentration may be a biomarker of endothelial dysfunction [112]. Fahs et al. have presented proof that DHA can alleviate the endothelial-function aberrations that are caused by a high-fat diet [113].

Some studies address the question which *ω*-3 PUFA has the strongest influence on the blood lipid profile. Both EPA and DHA effectively diminish the level of TG but differently affect the levels of HDL and LDL cholesterol [114,115]. Besides, concentrations of circulating DHA and EPA are inversely proportional to the prevalence of CVD [116].

**Docosapentaenoic acid (DPA) (C22:5)** is a PUFA of the *ω*-3 class and is an intermediate product between EPA and DHA [117]. In food sources, the DPA amount is too low. DPA is a precursor of a large class of lipid mediators (special proteins, resolvins, maresins, and isoprostanes) mainly taking part in the elimination of inflammation, with specific effects in contrast to other *ω*-3 PUFAs. The DPA is beneficial for the cardiovascular system and correlates with risk markers of metabolic diseases, especially with parameters of blood lipids, with platelet aggregation, and with insulin sensitivity [118].

Japanese researchers [119] have documented a significant correlation between nutritional DPA supplementation and lower prevalence of CVD. This finding has been confirmed in a control study with a six-year observation period, which also indicates that the link between DPA and CVD does not depend on other *ω*-3 FA.

Recent research further clarified which pathophysiological mechanisms of action of DPA may target CVD. A prospective cohort study suggests that the total concentration of *ω*-3 FA is associated with lower prevalence of congestive heart failure, with DPA lowering this parameter to 40% of the control level [120]. In another study, researchers investigated the impact of *ω*-3 PUFA on instability of the atherosclerotic plaque in a coronary artery [121]; low blood concentrations of DPA significantly correlated with plaques enriched with lipids. Those authors supposed that downregulation of DPA may promote the frequency of unstable plaque formation leading to the development of acute coronary syndrome and MI [121].

It is known that platelet aggregation is an early event in the development of thrombosis and is initiated by thromboxane A2. The EPA, DPA, and DHA attenuate the platelet aggregation stimulated by collagenic or arachidonic acid (ARA), depending on the dose. The DPA was found to be an almost tenfold more potent inhibitor than EPA. Further research on human whole blood has confirmed these data [122,123]. The absence of EPA, DPA, or DHA in the circulation may be an independent predictor and risk marker of CVD [124].

The Omega-3 Index may be the marker of choice for the 21st century because fulfills many of the criteria of a CVD risk factors [125]. Tribulova N. et al. [125] have studied the most Recent Data on the Omega-3 Index. Taken together, it appears that the omega-3 index may be a good candidate as a biomarker for assessing the risk of cardiac undesirable events. However, available data suggest that the anti-arrhythmic window for circulating *ω*-3 PUFAs is narrow. Thus, relatively high levels of free *ω*-3 PUFAs (e.g., due to infusion) may not always be associated with protection of the acutely injured heart. Nevertheless, monitoring of both the omega-3 index and plasma levels may reflect the actual status of the *ω*-3 PUFAs that should always be considered with respect to their effects.

## 5. Omega-6 Polyunsaturated Fatty Acids

These PUFA are less studied than *ω*-3 FA. Although most articles suggest that PUFA are overall effective against risk factors of CVD such as dyslipoproteinemias, hypertension, and atherosclerosis. It is feared that high levels of *ω*-6 PUFA compared to *ω*-3 PUFA may worsen cardiovascular risk by increasing inflammation. Therefore it is still debated whether the activities of *ω*-6 PUFA are pro- or anti-inflammatory. Some investigators have reported antagonistic effects of PUFA: Although *ω*-3 FA manifest themselves as cardioprotectors, *ω*-6 PUFA exert pro-inflammatory properties [126,127,128].

However, this view is now enjoys little to no direct support from studies in humans. In a recent Cochrane meta-analysis, which included 19 randomized controlled trials and 6461 participants, compared higher and lower omega intake-6 FA in adults with or without CVD, effects were evaluated for at least 12 months [129]. Authors found that increasing *ω*-6 PUFA may reduce risk of MI. Although the benefits of *ω*-6 PUFA remain unproven, increasing their intake may benefit people at high risk of developing MI. It has been shown that increasing *ω*-6 PUFA reduces serum total cholesterol over at least one year, but not other lipid fractions in the blood. In addition, in recent years, the complex biochemistry of the eicosanoids (and docosanoids and octadecanoids) has become clearer, so the class itself *ω*-6 PUFA can no longer be so simply regarded as pro-inflammatory [130].

These new findings began to erode the view that PUFA biology could be summed up in one simple the omega-6/omega-3 ratio. For ensuring the body has the proper level of *ω*-3 FA reactions and the possibility of good health, changes in the participation of *ω*-3 and *ω*-6 FAs in the Daily Nutritional Ration are important. According to the Polish Forum of Cardiovascular Disease Prophylactic Program, the proportion of *ω*-6 to *ω*-3 acids should be 4:1 [131]. Nevertheless, in the literature more and more often you can find a ratio of 10:1 and higher. Both the shortage and over-use of FA may be harmful to the human body. Therefore, we can assume that a higher inflammatory status can be seen in settings where the omega-6/omega-3 ratio are high, but the problem is not a large content of *ω*-6 FA, but rather the absence of *ω*-3 FA.

**Linoleic acid (LA) (C18:2)** is the most prevalent FA in phospholipids, in particular in cardiolipins. It constitutes ~10% of FA content of adipose tissue and more than 20% of all FA in human blood. LA is not synthesized in the mammalian (human) body. Substantial amounts are present in plant oils. Half of all cholesterol esters in the blood are formed from LA.

The main role of LA in the body (animals and humans) is a biochemical precursor of physiologically important long-chain PUFA, such as ARA, with subsequent synthesis of proinflammatory eicosanoids (prostaglandin E2), leukotriene B4, and thromboxane A4 [132,133,134]. Higher production of proinflammatory eicosanoids may lead to the emergence of other biomarkers of inflammation (e.g., interleukin 6, TNF-*α*, and C-reactive protein), which are linked with a higher risk of CVD. A high consumption of LA may cause the increased of ALA and total amounts of *ω*-3 EPA and DHA actually increased slightly [135]. On the other hand, consumption of LA as a nutritional supplement does not affect the concentrations of various circulating inflammatory biomarkers [136,137]. In addition, a number of clinical studies have been conducted that addressed the link between LA consumption and inflammation. For example, Ferrucci et al. [138] have noted that total concentrations of *ω*-6 PUFA in the blood are inversely proportional to blood concentrations of C-reactive protein and interleukins -1 and -6, and to the TNF-*α* level. According to Choo et al. [139], LA blood concentration inversely correlates with the size of very-LDL and LDL particles and directly correlates with HDL particle size. Available evidence from randomized controlled trials shows that replacement of saturated fat with linoleic acid effectively lowers serum cholesterol but does not support the hypothesis that this translates to a lower risk of death from coronary heart disease or all causes [140]. In a meta-analysis of 22 studies, including prospective cohort studies and case-control studies, concentrations of LA in the blood and tissue were found to inversely correlate with nonfatal endpoints of IHD [141].

***γ*-Linolenic acid (GLA) (C18:3)** is a PUFA whose chain is composed of 18 carbon atoms and three DB at positions 6, 9, and 12. The GLA is found in oils from various seeds but is usually taken as a nutritional supplement [142]. The GLA can stimulate the production of eicosanoids (in particular prostaglandin E2), exerts anti-inflammatory and anti-proliferative effects, and can lower the levels of lipids [143]. A possible indication for nutritional supplementation with GLA is a confirmed lower GLA content of phospholipids and of cholesterol esters in the blood of patients with hypertriglyceridemia or HC [144]. In a study by Schwab et al. [145] on people taking GLA as a nutritional supplement, there was a significant decrease in blood concentrations of TG, total cholesterol, and LDL, with upregulation of the HDL fraction.

Data regarding the influence of GLA on adverse cardiovascular events and mortality rates of CVD are virtually nonexistent. Das et al. [146] have found that GLA benefits blood vessels by preventing arterial hypertension and helps to prevent IHD complications by means of the blood-clotting system.

**Arachidonic acid (ARA) (C20:4)** is a 20-carbon compound with four DB at positions 5, 8, 11, and 14. The ARA is component of phospholipids, TG, and cholesterol esters. It constitutes 2% to 8% of the total amount of FA. Endogenous production of ARA mainly occurs because of its liberation from phospholipids of the cellular membrane. This process is catalyzed by enzymes of the phospholipase A2 superfamily [147]. Given that human food contains large amounts of LA, the amount of ARA almost always exceeds the level necessary for the maintenance of the balance among PUFA [148]. It is believed that ARA is a precursor of prostaglandins and many other eicosanoids [149], imparts flexibility and fluidity to cellular membranes, serves as a lipid secondary messenger in cellular signaling, acts as an inflammatory mediator, and causes vasodilatation [150]. Furthermore, ARA influences the activity of the cardiac proton pumps that make the main contribution to cardiomyocyte excitability.

The ARA can induce oxidative stress [151], which is an important factor of atherosclerosis pathogenesis [152]. A higher blood level of ARA is related to higher prevalence of arterial hypertension [153]. The research into the risk of atherosclerosis has revealed that lower LA levels and higher blood levels of ARA correspond to a higher risk of hypertension; moreover, ARA levels directly correlated with the body mass index and related metabolic syndrome [154,155,156].

On the whole, for atherosclerosis prophylaxis, the most important PUFA are essential FA of the *ω*-6 family. Their hypocholesterolemic activity is attributed primarily to a decrease in the concentration of LDL cholesterol and in the blood level of SFA as well as upregulation of LA, which undergoes oxidation more slowly than ARA does.

Finally, we summarized the data and presented it in summary Table 4 that reports positive and negative effects of different FA family on the cardiovascular system.

## 6. Conclusions

Consequently, the research into the influence of individual FA on the etiopathogenesis, diagnosis, prevention, risk assessment, and treatment of CVD is a promising rapidly developing field of biomedicine. The possible contradictions between experimental and clinical results depend on different concentrations of the studied drugs, their composition, sample size, and duration of observation. However, data on associations of various SFA and unsaturated FA with lipid–lipoprotein parameters and with inflammatory and oxidative markers of CVD are also intriguing and prompting more and more studies for clarification and elaboration of already known mechanisms underlying the influence of FA on the cardiovascular system.

## Figures and Tables

**Table 1:** The most physiologically important saturated fatty acids.

The Notation of Fatty Acid (Number of Carbon Atoms: Number π Bonds)	Trivial Name	Systematic Name (IUPAC)	Chemical Formula
12:0	Lauric	Dodecanoic	CH_3_–(CH_2_)_10_–COOH
14:0	Myristic	Tetradecanoic	CH_3_–(CH_2_)_12_–COOH
16:0	Palmitic	Hexadecanoic	CH_3_–(CH_2_)_14_–COOH
18:0	Stearic	Octadecanoic	CH_3_–(CH_2_)_16_–COOH
20:0	Arachidic	Eicosanoic	CH_3_–(CH_2_)_18_–COOH
22:0	Behenic	Docosanoic	CH_3_–(CH_2_)_20_–COOH
24:0	Lignoceric	Tetracosanoic	CH_3_–(CH_2_)_22_–COOH

**Table 2:** The most common monounsaturated fatty acids.

The Notation of Fatty Acid (Number of Carbon Atoms: Number π Bonds)	Trivial Name	Systematic Name (IUPAC)	Chemical Formula
12:1	Lauroleic	CIS-9-dodecenoic	CH_3_–CH_2_–CH=CH(CH_2_)_7_–COOH
14:1	Myristoleic	CIS-9-tetradecenoic	CH_3_–(CH_2_)_3_–CH=CH(CH_2_)_7_–COOH
16:1	Palmitoleic	CIS-9-hexadecenoic	CH_3_–(CH_2_)_5_–CH=CH–(CH_2_)_7_–COOH
18:1	Oleic	CIS-9-octadecenoic	CH_3_–(CH_2_)_7_–CH=CH–(CH_2_)_7_–COOH
18:1	Petroselinic	CIS-6-octadecenoic	CH_3_–(CH_2_)_10_–CH=CH–(CH_2_)_4_–COOH
	Vaccenic	CIS-11-octadecenoic	CH_3_–(CH_2_)_5_–CH=CH–(CH_2_)_9_–COOH
20:1	Gadoleic	CIS-9-eicosanoic	CH_3_–(CH_2_)_9_–CH=CH–(CH_2_)_7_–COOH
	Gondoic	CIS-11-eicosenoic	CH_3_–(CH_2_)_7_–CH=CH–(CH_2_)_9_–COOH
22:1	Erucic	CIS-13-docosanoic	CH_3_–(CH_2_)_7_–CH=CH–(CH_2_)_11_–COOH
	Cetoleinoic	CIS-11-docosanoic	CH_3_–(CH_2_)_5_–CH=CH_3_–(CH_2_)_11_–COOH
24:1	Nervonic	CIS-15-tetracosenoic	CH_3_–(CH_2_)_7_–CH=CH(CH_2_)_13_–COOH

**Table 3:** The most physiologically important (common) polyunsaturated fatty acids.

The Notation of Fatty Acid (Number of Carbon Atoms: Number π Bonds)	Trivial Name	Systematic Name (IUPAC)	Chemical Formula
**Dienoic Acids**			
18:2 (*ω*-6)	Linoleic	9,12-octadecadienoic	CH_3_–(CH_2_)_4_–CH=CH–CH_2_–CH=CH–(CH_2_)_7_–COOH
20:2 (*ω*-6)	Eicosadienoic	11,14-eicosadienoic	CH_3_–(CH_2_)_4_–CH=CH–CH_2_–CH=CH–(CH_2_)_9_–COOH
**Trienoic Acids**			
18:3 (*ω*-3)	*α*-linolenic	9,12,15-octadecatrienoic	CH_3_–CH_2_–CH=CH–CH_2_–CH=CH–CH_2_–CH=CH–(CH_2_)_7_–COOH
18:3(*ω*-6)	*γ*-linolenic	6,9,12-octadecatrienoic	CH_3_–(CH_2_)_4_–CH=CH–CH_2_–CH=CH–CH_2_–CH=CH–(CH_2_)_4_–COOH
20:3 (*ω*-6)	Eicosatrienoic	Eicosatrienoic-5,8,11	CH_3_–(CH_2_)_7_–CH=CH–CH_2_–CH=CH–CH_2_–CH=CH–(CH_2_)_3_–COOH
**Tetraenoic Acids**			
20:4 (*ω*-6)	Arachidonic	5,8,11,14-eicosatetraenoic	CH_3_–(CH_2_)_4_–CH=CH–CH_2_–CH=CH–CH_2_–CH=CH–CH_2_–CH=CH–(CH_2_)_3_–COOH
**Pentaenoic Acids**			
20:5 (*ω*-3)	Timnodonic	Eicosapentaenoic-5,8,11,14,17	CH_3_–CH_2_–CH=CH–CH_2_–CH=CH–CH_2_–CH=CH–CH_2_–CH=CH–CH_2_–CH=CH–(CH_2_)_3_–COOH
22:5 (*ω*-3)	Clupanodonic	7,10,13,16,19-docosapentaenoic	CH_3_–CH_2_–CH=CH–CH_2_–CH=CH–CH_2_–CH=CH–CH_2_–CH=CH–CH_2_–CH=CH–(CH_2_)_5_–COOH
**Hexaenoic Acids**			
22:6 (*ω*-3)	Cervonic	Docosahexaenoic-4,7,10,13,16,19	CH_3_–CH_2_–CH=CH–CH_2_–CH=CH–CH_2_–CH=CH–CH_2_–CH=CH–CH_2_–CH=CH–CH_2_–CH=CH–(CH_2_)_2_–COOH

**Table 4:** The influence of fatty acids on the cardiovascular system.

Fatty Acids	The Influence on the Cardiovascular System	Effect of Increasing the Level	Effect of Reducing the Level
Myristic acid (C14:0)	-	↑ the risk of developing IHD; ↑ total cholesterol concentration [32,39]	
Palmitic acid (C16:0)	-	↑ production of pro-inflammatory cytokines and oxidants; promotes inflammation and the development of CVD [31,32,34,35,36,37]	↓ LDL, glucose, arterial blood pressure normalizes [30]
Stearic acid (C18:0)	±	not correlate with a higher risk of IHD and MI; induces apoptosis and necrosis of endothelial cells; ↑ the risk of developing IHD [27,44,45,46,47,48]	does not have a significant influence on lipid metabolism [10,41]
Palmitoleic acid (C16:1)	±	↓ cholesterol and TG concentrations; ↑ HDL cholesterol concentrations [56,57]; ↑ the risk of developing IHD [32]	
Oleic acid (C18:1)	±	can improve the blood lipid profile [65]; ↓ the risk of atherosclerosis [69]; ↑ TG concentrations; ↑ markers of inflammation; ↑ the risk of developing heart failure [36,71]	
Linoleic acid (C18:2)	+	↓ risk developing IHD and death from IHD [140,141]; does not affect the concentrations of inflammatory biomarkers [146,147]	↑ risk of arterial hypertension [154,155,156]
*α*-Linolenic acid (C18:3)	+	↓ the risk of death from IHD; ↓ the levels of LDL and total cholesterol; ↑ the levels of HDL [80,81,82];	↑ development of IHD [88]
*γ*-Linolenic acid (C18:3)	+	↓ concentrations of TG, total cholesterol and LDL; ↑ of the HDL concentrations [145]; ↓ development of arterial hypertension [146]	↑ TG concentrations [144]
Arachidonic acid (C20:4)	-	↑ of biomarkers of inflammation; risk of CVD [132,133,134]; ↑ risk of arterial hypertension [153,154,155,156]; ↑ the risk of atherosclerosis [151,152];	
Eicosapentaenoic acid (C20:5)	+	can improve the blood lipid profile [90,107]; ↓vascular inflammation; ↓ risk developing of major ischemic events, and death from IHD [91,107]	↑ risk developing of IHD [124]
Docosapentaenoic acid (C22:5)	+	↓ prevalence of CVD; ↓ developing of congestive heart [119,120]	↑ developing of plaques enriched with lipids; promote the frequency of unstable plaque formation leading to the development of acute coronary syndrome and MI [121]; ↑ risk developing of IHD [124]
Docosahexaenoic acid (C22:6)	+	↓ concentrations of TG; ↑ the HDL concentrations [109]; ↓ inflammation markers [110,111]	↑ endothelial dysfunction [112,113]; ↑ risk developing of IHD [124]

«-»—the adverse effect, «+»—the beneficial effect, «±»—the questionable impact, «↓»—the decrease, «↑»—the increase, IHD—ischemic heart disease, CVD—cardiovascular disease, LDL—low-density lipoproteins, HDL—high-density lipoprotein cholesterol, MI—myocardial infarction, TG—triglyceride.
